# Immune Thrombocytopenia in a Child with Neuroblastoma

**DOI:** 10.1155/2017/1329489

**Published:** 2017-09-14

**Authors:** Hasan Tarkan Ikizoglu, Inci Ayan, Fatma Tokat, Tulay Tecimer, Gonca Topuzlu Tekant

**Affiliations:** ^1^Department of Pediatrics, Acibadem University Medical Faculty, Istanbul, Turkey; ^2^Department of Pediatrics, Acibadem Maslak Hospital, Istanbul, Turkey; ^3^Department of Pathology, Acibadem Maslak Hospital, Istanbul, Turkey; ^4^Department of Pediatric Surgery, Cerrahpasa Medical Faculty, Istanbul University, Istanbul, Turkey

## Abstract

Thrombocytopenia is a frequent finding in patients with solid tumors. It is usually caused by bone marrow infiltration or by myelosuppression due to anticancer therapy; however immune thrombocytopenia (ITP) associated with solid tumors is rare. Neuroblastoma is the most common extracranial solid tumor in children. Here we report the case of a two-year-nine-month-old patient with adrenal neuroblastoma who presented with ITP. Paraneoplastic ITP was considered in the differential diagnosis. Bone marrow infiltration and other causes of thrombocytopenia were excluded and the patient was treated with intravenous immunoglobulin and tumor resection. Platelet count increased rapidly after surgery and complete remission of ITP was achieved.

## 1. Introduction

Thrombocytopenia is a frequent finding in patients with cancer and is usually caused by bone marrow involvement of malignant cells or by toxicity from anticancer therapy. Rarely immune thrombocytopenia (ITP) is reported at the time of diagnosis. Although this condition could represent incidental finding of two common pathologies, paraneoplastic processes should also be considered. Immune thrombocytopenia has been reported as a paraneoplastic syndrome with several tumors [1–3]. However, to our knowledge it has not been reported previously in patients with neuroblastoma.

Neuroblastoma is the most common extracranial solid tumor in children [4]. Neuroblastoma can arise anywhere along the sympathetic nervous system. The majority of tumors (65%) arise in the abdomen, with over half of these arising in the adrenal gland. Clinical presentation of neuroblastoma is dependent upon the site of tumor origin, disease extent, and the presence of paraneoplastic syndromes [5].

Herein, we report a case of neuroblastoma presenting with ITP and we discuss the probability of paraneoplastic thrombocytopenia.

## 2. Case Presentation

A 2-year-9-month-old male patient with a left adrenal mass detected by abdominal ultrasonography was referred to our institution. His chief complaints were paleness, abdominal pain, and back pain of one-week duration. Fever or findings consistent with viral infection, history of vaccination, and drug use during the previous weeks were not reported. His previous medical history and family history were unremarkable.

He had mild pallor; his vital signs, growth, and development were normal. Physical examination revealed a mass at the left upper quadrant of the abdomen and several ecchymotic lesions measuring 1 to 1.5 cm in diameter, on the pretibial areas. Hepatosplenomegaly and lymphadenopathy were not detected. Complete blood count (CBC) revealed thrombocytopenia and mild anemia with hemoglobin (Hb): 9.6 g/dl, MCV: 75.8 fL, platelet (PLT): 53000/mm^3^, mean platelet volume (MPV): 9,1 fL, and white blood cell: 10740/mm^3^. Lactate dehydrogenase was 392 IU/L (normal range ≤ 345); other laboratory tests including renal and liver function tests, serum electrolytes, prothrombin time, activated partial thromboplastin time, fibrinogen, and ferritin were within normal range. The serology tests for HIV, Hepatitis B, and Hepatitis C were negative.

**Figure 1:**
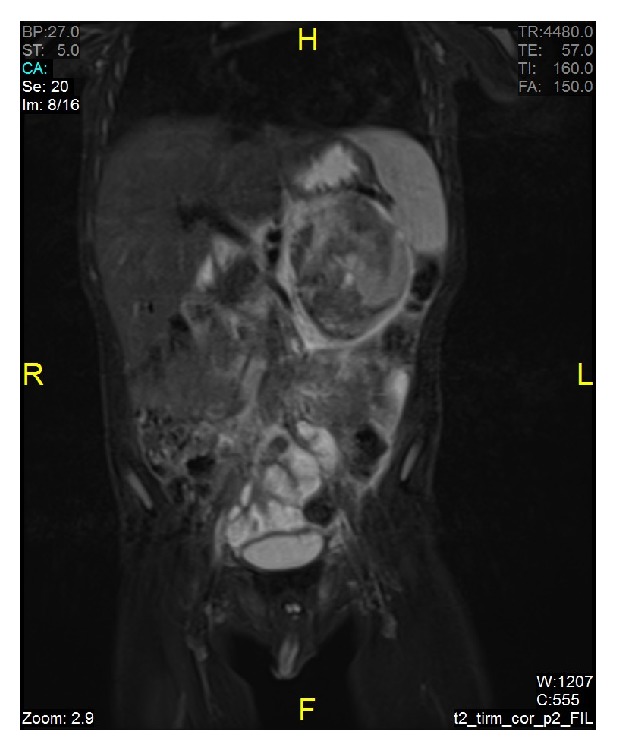
Heterogeneous mass at the left adrenal gland with smooth borders, containing cystic and hemorrhagic areas.

Abdominal MRI was performed. A heterogeneous mass with smooth borders, containing cystic and hemorrhagic areas, measuring 59 × 60 × 74 mm was detected at the left adrenal gland. The tumor did not cross the midline and no vascular invasion, lymphadenopathy, or metastases to other abdominal organs were detected with MRI (Figure 1). Neuroblastoma was considered.

Patient was admitted to hospital for further evaluation and surgery. Neuron specific enolase was found to be 26 ng/ml (normal range ≤ 20); serum catecholamines were within normal range. Tumor metastasis was not observed in I-131 Metaiodobenzylguanidine and Technetium-99 m scan.

**Figure 2:**
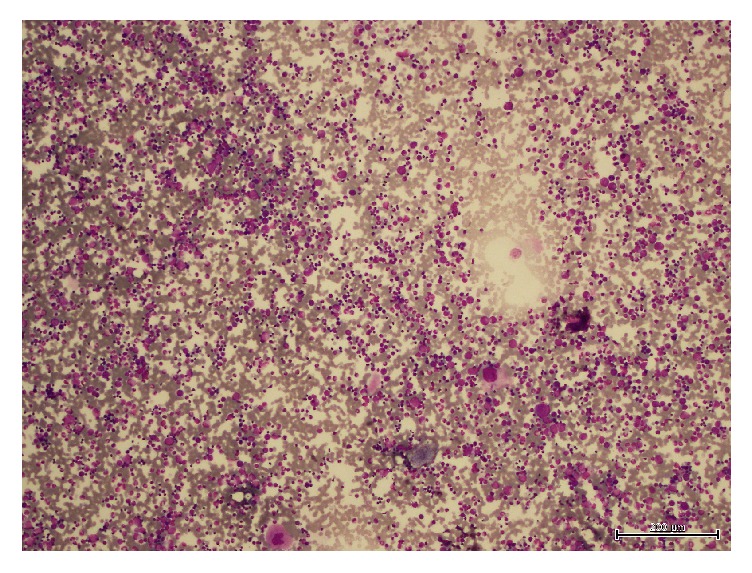
Normocellular heterogeneous bone marrow demonstrating increased number of mature and immature megakaryocytes.

CBC was repeated and showed a platelet count of 26000/mm^3^. Bilateral bone marrow biopsy and aspiration were performed for tumor staging and for investigating the etiology of the thrombocytopenia. Cytomorphologic examination revealed normocellular bone marrow with continuous maturation in erythroid, myeloid, and megakaryocytic elements. The number of megakaryocytes was increased. Young forms of megakaryocytes and rare micromegakaryocytes were observed as well as mature forms. There were no tumor cells, pseudorosettes, storage cells, and/or erythrophagocytosis in the bone marrow; thus an immune thrombocytopenia was considered (Figure 2).

**Figure 3:**
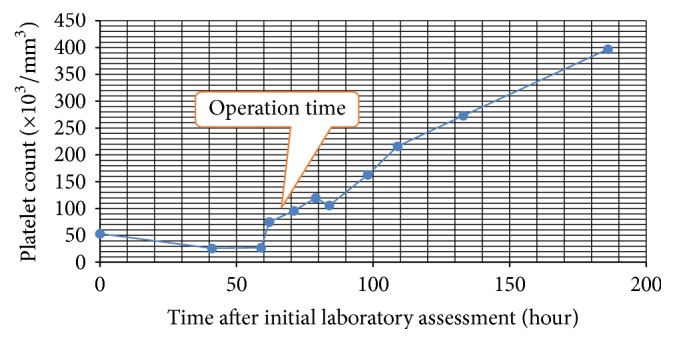
Platelet count during the follow-up.

The patient was treated with 0.8 g/kg of intravenous immunoglobulin (IVIG) and subsequently transfused with platelets in order to maintain an acceptable level of thrombocytes for diagnostic and therapeutic surgery [6]. An increase in platelet count was achieved from 26000 to 75000/mm^3^ at the third hour following IVIG and platelet transfusion. The patient underwent surgery and a 70 × 65 × 45 mm left adrenal mass was totally resected. The operation was completed without any major bleeding and/or other complications. Immediately after the surgery, platelet count was detected 96000/mm^3^; it was raised to normal levels at the 36th hour after surgery and remained stable afterwards which supported the diagnosis of ITP (Figure 3).

**Figure 4:**
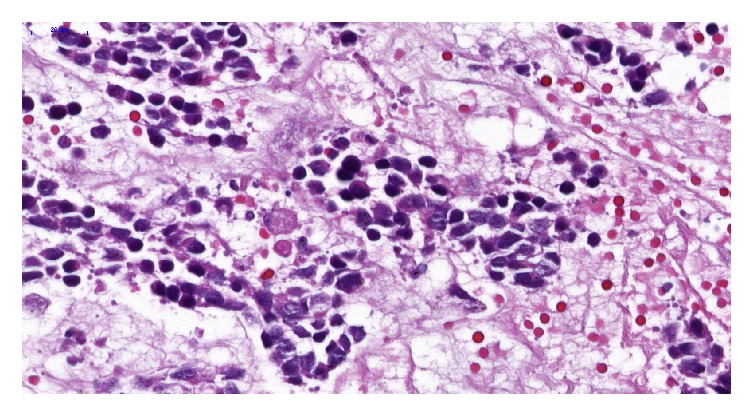
Neuroblastoma (Schwannian-stroma-poor), differentiating subtype, composed predominantly of differentiating neuroblasts with moderate to abundant acidophilic cytoplasm and enlarged, eccentric nucleus with vesicular chromatin and single prominent nucleolus. Abundant neuropil is also present.

Tumor histopathology revealed a Schwannian-stroma-poor, differentiating neuroblastoma with low mitotic rate and low mitosis-karyorrhexis index (MKI < 100/5000 cells) [7] (Figure 4). The resection margins were free of tumor cells.

The patient was diagnosed as stage 1 neuroblastoma according to the International Staging System [8] and immune thrombocytopenia. According to the neuroblastoma treatment protocol the patient was not given any adjuvant chemotherapy and/or radiotherapy and a follow-up program was started. During the six-year follow-up recurrence of ITP was not observed.

## 3. Discussion

Thrombocytopenia in cancer patients is induced by several mechanisms: (1) decrease in platelet production caused by bone marrow infiltration or by bone marrow suppression due to cytotoxic drugs, radiotherapy, or infections, (2) increased platelet destruction due to immune pathologies or disseminated intravascular coagulopathy (DIC), (3) platelet sequestration as a result of splenic metastasis. Immune thrombocytopenia is mainly a diagnosis of exclusion and all other causes for thrombocytopenia must be ruled out in order to confirm the diagnosis. ITP can be either primary (idiopathic) or secondary. Although ITP with tumors may be a coincidental finding, it has been described as a paraneoplastic syndrome of several tumors [1–3].

The association between ITP and lymphoid neoplasms, particularly chronic lymphoid leukemia and Hodgkin's disease, is well described [1, 9]. The prevalence of ITP in Hodgkin's disease is 0.79% [1] and 0.76% in non-Hodgkin's lymphoma [10]. However, the association with solid tumors is less recognized. ITP has been reported to present with various types of solid tumors including breast, lung, kidney, germ cell, and ovary cancer [11].

Paraneoplastic syndromes are disorders that are not directly related to the growth, invasion, or metastasis of the tumor but occur as a result of autonomous secretion of hormones, peptides, or cytokines by the tumor. The major paraneoplastic syndromes associated with neuroblastomas are opsoclonus-myoclonus-ataxia syndrome (OMAS), which is believed to have an autoimmune pathogenesis [4, 12] and intractable watery diarrhea due to the autonomous tumor secretion of vasoactive intestinal peptide (VIP) [4, 13]. To date, paraneoplastic ITP diagnosed at presentation in patients with neuroblastoma has not yet been reported.

In patients with neuroblastoma, although very frequent during chemotherapy and/or radiotherapy, thrombocytopenia at diagnosis is usually related to bone marrow infiltration by the tumor cells, which results in stage IV disease. In the present case, thrombocytopenia was detected at initial evaluation before the commencement of therapy. There was no evidence of infection or DIC. Splenic involvement that could result in thrombocyte sequestration was ruled out by abdominal MRI. Mean platelet volume was normal. Bilateral bone marrow aspiration and biopsy were performed and tumor involvement was ruled out. Bone marrow aspiration revealed normocellular bone marrow with no tumor cells, both young forms and mature megakaryocytes were observed and the number of megakaryocytes was increased suggesting an autoimmune process accompanying neuroblastoma rather than bone marrow infiltration. Although tests for platelet antibodies could not be performed we concluded that thrombocytopenia in this case was immunologic in origin after reviewing his clinical, laboratory, and imaging findings. Since ITP may occur in isolation (primary) or in association with other disorders (secondary), secondary causes were investigated [6]. The patient did not have fever or other findings consistent with viral infection during the initial examination or during the previous weeks. The use of any medication, recent vaccination, and blood transfusion were denied. HIV and hepatitis infection were ruled out with serological tests. There was no evidence of rheumatologic disease. Therefore we concluded that immune thrombocytopenia in this patient was either primary ITP or secondary ITP associated with neuroblastoma. Although neuroblastoma and primary ITP could occur coincidentally, rapid recovery of thrombocytopenia after tumor resection may support the diagnosis of paraneoplastic ITP.

The mechanism of immune thrombocytopenia associated with tumors remains to be elucidated. Immune dysregulation occurring in the setting of cancer is thought to result in increased platelet destruction [9]. Autoantibodies against platelet antigens have been demonstrated in some patients with ITP associated with lymphomas and solid tumors; however they were not shown to be specific for paraneoplastic ITP [2, 3, 14, 15].

Treatment of paraneoplastic ITP associated with solid tumors included various combinations of corticosteroids, splenectomy, IVIG, and interferon in addition to primary treatment of the underlying malignancy with surgery, chemotherapy, and/or radiotherapy. Complete remission of ITP after curative surgery was generally low and additional therapeutic agents were needed [11]. In the present case IVIG and subsequent platelet transfusion were used for managing ITP because rapid elevation of platelet count was essential for performing surgery. Platelet count was raised to normal levels after the resection of tumor without requiring any other supportive treatment.

In conclusion the aim of this case presentation is to emphasize that bone marrow infiltration is not the only cause of thrombocytopenia at diagnosis in neuroblastoma. Although rarely, an immune thrombocytopenia either primary or paraneoplastic has to be taken into consideration. A prompt and appropriate intervention for thrombocytopenia may allow the specific oncological treatment.
